# Treatment of Diarrhœa in Phthisis

**Published:** 1854-10

**Authors:** 


*Treatment of Diarrhœa in Phthisis.*—Among the out-patients attending at the City Hospital for Chest Diseases, cases of diarrhœa have been unusually frequent for the last two months, in consequence, no doubt, of the epidemic influence now prevailing. Those occurring to the subjects of phthisical disease have, in many instances, been more difficult to control than usual. The cases not being of the acute or choleraic type, the sulphuric acid treatment has been tried in but few instances. The remedy chiefly relied upon has been opium, exhibited in one or other of the following forms:—In mild cases of relaxed bowels, attended with much irritability of the stomach, the diarrhœa being liable to be set up again by every slight dietetic cause, ℞. Bism. trisnitr. gr. x., mucilaginis ʒij. vini. opii. mx., acid. hydrocy. dil. mij., aq. pur. ℥j. Ft. haust. ter die sumend. This mixture, continued for a week or two, has often sufficed to relieve the condition alluded to, and enable the stomach to tolerate, not only ordinary plain food, but even the cod-liver oil. In more severe, suddenly occurring cases, ℞. Pulv. kino co. gr. x., o. n. vel o. n. et m. sum. Patients liable to attacks of sudden and severe diarrhœa have been directed to keep these powders on hand, and take them at once on a re-appearance of the symptom. In many cases of the latter class, Dr. Peacock prefers to the kino a pill containing the acetate of lead and opium (gr. ii., and gr. ss.) given either every night or twice in the day, as circumstances indicate. The employment of the emulsion of mutton suet and milk in cases of irritable bowels is a very favorite practice at this Hospital, and, in the milder class, frequently suffices without resort to other remedies. The patient is directed to procure fresh mutton suet, cut it very fine, tie it loosely in a coarse muslin bag, and let it gently simmer in milk for a few minutes, after which a quantity of the best cinnamon is to be grated in. The quantity taken may depend upon the patient's palate, and it is generally well relished. The cinnamon probably exerts, as an astringent, an important influence, and should never be omitted.

